# Antineoplastic Activity of the Combination Loratadine–Simvastatin–Gefitinib in HPV-Positive Cervical Cancer Cells: In Vitro and In Vivo Studies

**DOI:** 10.3390/cancers18162589

**Published:** 2026-08-12

**Authors:** Tania Raya-Bahena, Rene M. Rivera-Escobar, Elisabeth Hernández-Gallegos, Javier E. Jiménez-Salazar, Pablo Damián-Matsumura, Janice García-Quiroz, Javier Camacho

**Affiliations:** 1 Departamento de Farmacología, Centro de Investigación y de Estudios Avanzados del Instituto Politécnico Nacional, Av. IPN 2508, Mexico City 07360, Mexico; tania.raya@cinvestav.mx (T.R.-B.); rene.rivera@cinvestav.mx (R.M.R.-E.); lishergallegos@yahoo.com (E.H.-G.); 2 Department of Biology of Reproduction, Biological Sciences and Health Division (DCBS), Autonomous Metropolitan University (UAM), Mexico City 09310, Mexico; bioquimicajejs@hotmail.com (J.E.J.-S.); pdamian@izt.uam.mx (P.D.-M.); 3 Departamento de Biología de La Reproducción Dr. Carlos Gual Castro, Instituto Nacional de Ciencias Médicas y Nutrición Salvador Zubirán, Av. Vasco de Quiroga No. 15, Belisario Domínguez Sección XVI, Tlalpan, Mexico City 14080, Mexico; janice.garciaq@incmnsz.mx

**Keywords:** cervical cancer, drug repurposing, loratadine, simvastatin, gefitinib, cisplatin, HPV, EGFR, apoptosis, combination therapy

## Abstract

**Background/Objectives**: Cervical cancer (CC) is the most clinically significant HPV-associated malignancy. Platinum-based chemotherapies produce drug resistance and serious adverse effects. Drug repurposing and combinations are promising approaches to improve the antitumor response. Here, we evaluated the antineoplastic potential of loratadine and simvastatin alone and in combination with cisplatin and gefitinib in HPV-positive CC cells. **Methods**: HeLa and SiHa CC cells were treated with loratadine, simvastatin, cisplatin, gefitinib, or their combinations. Metabolic activity was assessed using the MTT assay to determine drug inhibitory concentrations (IC_20_, IC_50_) from concentration–response curves. Apoptosis was assessed by Annexin V-FITC/PI flow cytometry. Clonogenic survival and migratory capacity were evaluated using colony formation and wound-healing assays, respectively. Tumor formation was evaluated in vivo using the chick chorioallantoic membrane model. **Results**: Metabolic activity decreased in a concentration-dependent manner following drug treatment in both cell lines. Several combination regimens at IC_20_ significantly improved reductions in metabolic activity and increases in apoptosis compared with monotherapies or two-drug combinations. Notably, some three-drug combinations, with or without cisplatin, had similar effects to the quadruple regimen. Combination treatments also reduced clonogenic survival and migratory capacity, as well as tumor formation and cancer cell dissemination in vivo. **Conclusions**: Drug combinations at relatively low concentrations (IC_20_) significantly enhanced the anticancer effects on CC cells in in vitro and in vivo settings. These findings support the potential of drug repurposing and combination strategies as promising approaches to decrease adverse side effects while improving therapeutic efficacy for the benefit of CC patients.

## Simple Summary

Cervical cancer (CC) is the most clinically significant HPV-associated malignancy, leading to thousands of deaths, especially in developing countries. Although cisplatin-based chemotherapy is the first-line treatment, the development of resistance and severe side effects frequently limits its use. Therefore, identifying safer and more effective therapeutic strategies is urgently needed. In this study, we evaluated the antitumor potential of the antihistamine loratadine and the cholesterol-lowering drug simvastatin, both as monotherapy and in combination with cisplatin and gefitinib in HPV-positive CC cells. Our findings showed that several drug combinations reduced cancer cell growth, induced cell death, inhibited cell migration, and decreased tumor formation. These results support drug repurposing and combination strategies as potential therapeutic approaches for CC treatment.

## 1. Introduction

Persistent high-risk human papillomavirus (HR-HPV) infection is a major oncogenic risk factor for multiple epithelial malignancies in the genital, anal, and throat areas. HR-HPV accounts for approximately 1,505,394 cancer cases and contributes to nearly 7.5% of the global cancer burden in 2022 [1].

Cervical cancer (CC) is the most clinically significant HPV-associated malignancy, with approximately 660,000 new cases and 350,000 deaths reported annually [2]. Globally, CC ranks as the fourth most common cancer among women, with nearly 94% of CC-related deaths occurring in low- and middle-income countries. Comorbidities such as HIV infection, as well as gender inequality, poverty, and limited access to vaccination, screening, and treatment services, are more frequent in such countries [3,4]. For example, in Mexico, CC is the second leading cause of cancer among women, with an estimated 13 deaths per day [5].

This well-established causal relationship between persistent HR-HPV infection and cervical carcinogenesis is the basis of current prevention strategies, including HPV vaccination and screening programs, which are the most cost-effective interventions for reducing disease burden globally [6].

Despite these prevention efforts, a substantial number of women continue to develop advanced, recurrent, or metastatic CC, for which the 5-year overall survival rate remains below 20%. At this stage of the disease, treatment options remain limited and mostly palliative [2].

Platinum- and taxane-based regimens are the first-line treatment for CC, but recurrence remains frequent, and overall survival remains poor [2,7]. Despite its clinical relevance and wide use, cisplatin treatment is frequently associated with severe adverse effects, including nephrotoxicity, ototoxicity, hepatotoxicity, and neurotoxicity [8]. Furthermore, the development of chemoresistance during treatment significantly limits the long-term effectiveness of these treatments.

These challenges underscore the need for more effective and better-tolerated therapeutic approaches. Consequently, drug repurposing and combination strategies have emerged as promising approaches to improve efficacy, overcome resistance, and reduce chemotherapy-associated toxicity in cancer patients. In this context, several molecular oncogenic targets and repurposed agents have shown promising antineoplastic activity. Epidermal growth factor receptor (EGFR) is frequently overexpressed in several cancers, including CC, and has been associated with poor clinical outcomes, making it a promising therapeutic target [9,10]. Gefitinib, a small-molecule EGFR tyrosine kinase inhibitor, has demonstrated antitumor activity in CC by inhibiting cell proliferation and epithelial–mesenchymal transition (EMT) and inducing apoptosis, both as a monotherapy and in combination with other chemotherapeutic agents [11,12,13,14,15]. However, the clinical efficacy of EGFR-targeted therapies remains limited by intrinsic and acquired resistance mediated through compensatory oncogenic pathways, highlighting the need for rational combination strategies targeting broader and more diverse anticancer molecular mechanisms.

Lipophilic statins block the mevalonate pathway and, beyond their lipid-lowering effects, exhibit antineoplastic properties by inhibiting proliferation, migration, invasion, and angiogenesis and promoting apoptosis in multiple cancer models [16,17]. Additionally, clinical and observational studies have associated statin use with improved survival and therapeutic responses in several CC malignancies [18,19,20,21,22,23]. These findings support their potential use as adjuvant agents in CC treatment. Additionally, simvastatin has been shown to enhance the efficacy of gefitinib in gefitinib-resistant non-small cell lung cancer (NSCLC) by increasing response rates and progression-free survival [24].

Loratadine, a widely used antihistamine, has demonstrated antineoplastic activity in multiple cancer types by inducing apoptosis, autophagy, and cell cycle arrest and inhibiting EMT and angiogenesis [25,26,27,28]. Moreover, preclinical studies suggest that loratadine may potentiate the efficacy of conventional anticancer therapies through several mechanisms, leading to enhanced apoptosis and reduced proliferative capacity [29,30].

Given the molecular complexity of cervical carcinogenesis, we selected clinically available drugs with diverse mechanisms of action that have been shown to improve clinical outcomes in several cancers, as described above. Therefore, we hypothesized that the combinations of these drugs would enhance antitumor activity. Because these drugs target several different pathways involved in cervical carcinogenesis, their diverse molecular mechanisms of action could enhance anticancer effects. In turn, the involvement of several pathways may help to avoid drug resistance, which may be of interest to oncologists. To our knowledge, this is the first study to comprehensively evaluate this drug repurposing and combination approach in HPV-positive cervical cancer using in vitro and in vivo CAM models. Our findings provide preclinical evidence that clinically available drugs are potentially translatable therapeutic approaches for HPV-associated cervical cancer.

## 2. Materials and Methods

### 2.1. Cell Lines and Drugs

The CC cell lines HeLa and SiHa were obtained from the American Type Culture Collection (ATCC, Manassas, VA, USA). Cells were cultured according to the manufacturer’s instructions and maintained under controlled conditions at 37 °C, 5% CO_2_, and 95% humidity. Cell lines were maintained in Dulbecco’s Modified Eagle Medium (DMEM) supplemented with 10% fetal bovine serum (FBS) and 1% antimycotic/antibiotic solution. For the HeLa cell line, glutamine (2 mM) and pyruvic acid (2 mM) were additionally supplemented. All culture reagents were acquired from Gibco (Thermo Fisher Scientific, Waltham, MA, USA). All drugs used in this study were purchased from Sigma-Aldrich Co. (St. Louis, MO, USA). Loratadine, simvastatin, and gefitinib were dissolved in dimethyl sulfoxide (DMSO) as the vehicle, whereas cisplatin was prepared in phosphate-buffered saline (PBS). All solutions were prepared using sterile reagents under aseptic conditions. The maximum final DMSO concentration in the vehicle control was 0.12% (*v*/*v*), corresponding to the concentrations of loratadine, simvastatin, and gefitinib.

### 2.2. Metabolic Activity Assay

Metabolic activity was evaluated as an indirect indicator of cell proliferation using the MTT (3-(4,5-dimethylthiazol-2-yl)-2,5-diphenyltetrazolium bromide) reduction assay, in which yellow MTT is converted to formazan by mitochondrial activity. HeLa and SiHa cells were seeded in 96-well plates at densities of 2000 and 3000 cells per well, respectively, and incubated for 24 h. Then, cells were treated for 72 h with a concentration range of loratadine (1–50 µM), simvastatin (10–70 µM), gefitinib (2.5–50 µM), cisplatin (HeLa: 0.10–15 µM; SiHa: 0.50–30 µM), or their respective vehicles (DMSO or PBS), with treatment refreshment at 48 h. Cells were then incubated with an MTT solution (0.5 mg/mL) for 4 h. Subsequently, 10% SDS in 0.01 M HCl was added, and the plates were incubated overnight.

Absorbance was measured at 595 and 690 nm using a Multiskan SkyHigh microplate spectrophotometer (Thermo Fisher Scientific, Waltham, MA, USA). Drug combination studies were performed following the same procedure.

The inhibitory concentration (IC) values were determined from concentration–response curves generated by the MTT assays for each drug. An IC value is defined as the concentration required to inhibit cellular metabolic activity by a defined percentage relative to the maximal effect within the tested concentration range. IC_20_ and IC_50_ values were obtained by analyzing the concentration–response curves of each drug in the MTT assays and fitting the data using a nonlinear regression model with GraphPad Prism (v10.0, GraphPad Software, San Diego, CA, USA).

### 2.3. Pharmacological Interaction Analysis

Drug interactions were evaluated using Bliss independence and highest single agent (HSA) models based on metabolic activity inhibition. Bliss excess values >0 indicate effects greater than expected, values = 0 indicate agreement with the expected effect, and values <0 indicate effects lower than expected. HSA scores >0 indicate that the drug combination outperformed the most active single agent, values = 0 indicate no additional effect, and values <0 indicate a lower effect than the most active single agent [31,32].

### 2.4. Flow Cytometry Analysis of Apoptosis

Apoptosis was assessed using the Annexin V-FITC kit (Thermo Fisher Scientific, USA). HeLa and SiHa CC cells were seeded in 60 mm Petri dishes at densities of 70,000 and 105,000 cells, respectively, and incubated for 72 h with vehicle, loratadine, simvastatin, gefitinib, and cisplatin, either as monotherapies or in double, triple, and quadruple combination treatments. Camptothecin and methanol served as positive controls for apoptosis and necrosis induction, respectively, and were added 24 h before the end of the incubation period. Samples were analyzed by flow cytometry using a CYAN ADP flow cytometer (Dako, Glostrup, Denmark). Cellular debris was excluded by FSC/SSC gating, and fluorescence compensation and quadrant settings were established using unstained and single-stained (Annexin V-FITC and PI) controls. Approximately 10,000 events were acquired per sample. The assay was performed in three independent biological experiments using different cell passages, each analyzed in duplicate.

### 2.5. Colony Formation Assay

This assay was performed to evaluate cell survival. HeLa cells were seeded in 60 mm Petri dishes at a density of 250 cells per plate, allowing isolated colony growth from individual cells. After 24 h, cells were incubated for 72 h at 37 °C in culture medium alone, in the presence of vehicle, individual drugs, or selected drug combinations. Subsequently, in the absence of treatments, cells were allowed to grow for an additional 7 days. After this period, cells were fixed with absolute ethanol for 15 min and stained with 1% crystal violet for an additional 15 min. Finally, plates were washed with water, and colonies were counted.

### 2.6. Wound-Healing Assay

Wound-healing assays were performed by culturing 1 × 10^6^ SiHa cells in 35 mm culture dishes and incubating for 24 h until a highly confluent monolayer was formed. A linear scratch was performed using a 200 µL pipette tip with constant pressure and direction. Cell rests were removed by washing with PBS, and cells were subsequently treated with either monotherapies or combination treatments. To avoid cell proliferation, cells were treated with cytarabine (Ara-C, 5 nM). Images of the same field were captured at 0, 24, and 48 h after wound generation. Images obtained in the wound-healing assay were quantified using ImageJ software (v1.54d, National Institutes of Health, Bethesda, MD, USA) by measuring the wound area at 0, 24, and 48 h and calculating the percentage of the wound relative to the initial wound area.

### 2.7. In Vivo Tumor Growth and Cell Spreading: The Chick Chorioallantoic Membrane (CAM) Assay

Specific pathogen-free (SPF) fertilized chicken embryos obtained from Sanfer^®^ (Mexico City, Mexico) were used for the in vivo chick CAM assay and maintained under controlled conditions at 37 °C and 95% humidity. Fertilized eggs were randomly assigned to nine experimental groups (2–3 embryos per group per experiment), and three independent experiments were performed. Non-viable embryos were excluded from the final analysis.

SiHa cells were cultured at a confluence of 1 × 10^6^ in 100 mm culture dishes and pretreated for 72 h with the corresponding monotherapies or selected combination regimens. Subsequently, a small opening was made in the eggshell to access the CAM. Approximately 1 × 10^6^ of the post-treated cells were injected into the chorioallantoic vein on embryonic day 15 (ED15). Embryos were euthanized on ED19 before hatching to assess metastatic dissemination. The heart, lungs, and cloaca were collected for further analysis. This study was conducted within an institutional research project reviewed by the Research Committee and approved by the Divisional Council of the Division of Biological and Health Sciences at Universidad Autónoma Metropolitana, Iztapalapa Unit (Approval No. CD.CBS.026.2023). In addition, concerning committee approval, our research at ED19 is in accordance with the current expert consensus on CAM model studies published in this journal [33].

### 2.8. Statistical Analysis

Statistical analyses were performed using GraphPad Prism software, and data are presented as mean ± SD. MTT assays were performed in three independent biological experiments with at least eight technical replicates, and concentration–response curves were fitted using nonlinear regression (four-parameter logistic model) to estimate IC_20_ and IC_50_ values with their corresponding 95% confidence intervals (95% CIs). Apoptosis, colony formation, and CAM assays were performed using three, four, and three independent biological experiments with three, four, and two to three technical replicates, respectively. Differences among groups were analyzed using one-way ANOVA followed by Tukey’s multiple-comparison post hoc test. A *p*-value < 0.05 was considered statistically significant.

## 3. Results

### 3.1. Loratadine, Simvastatin, Cisplatin, and Gefitinib Decrease Metabolic Activity in HeLa and SiHa CC Cells in a Concentration-Dependent Manner

**Figure 1:**
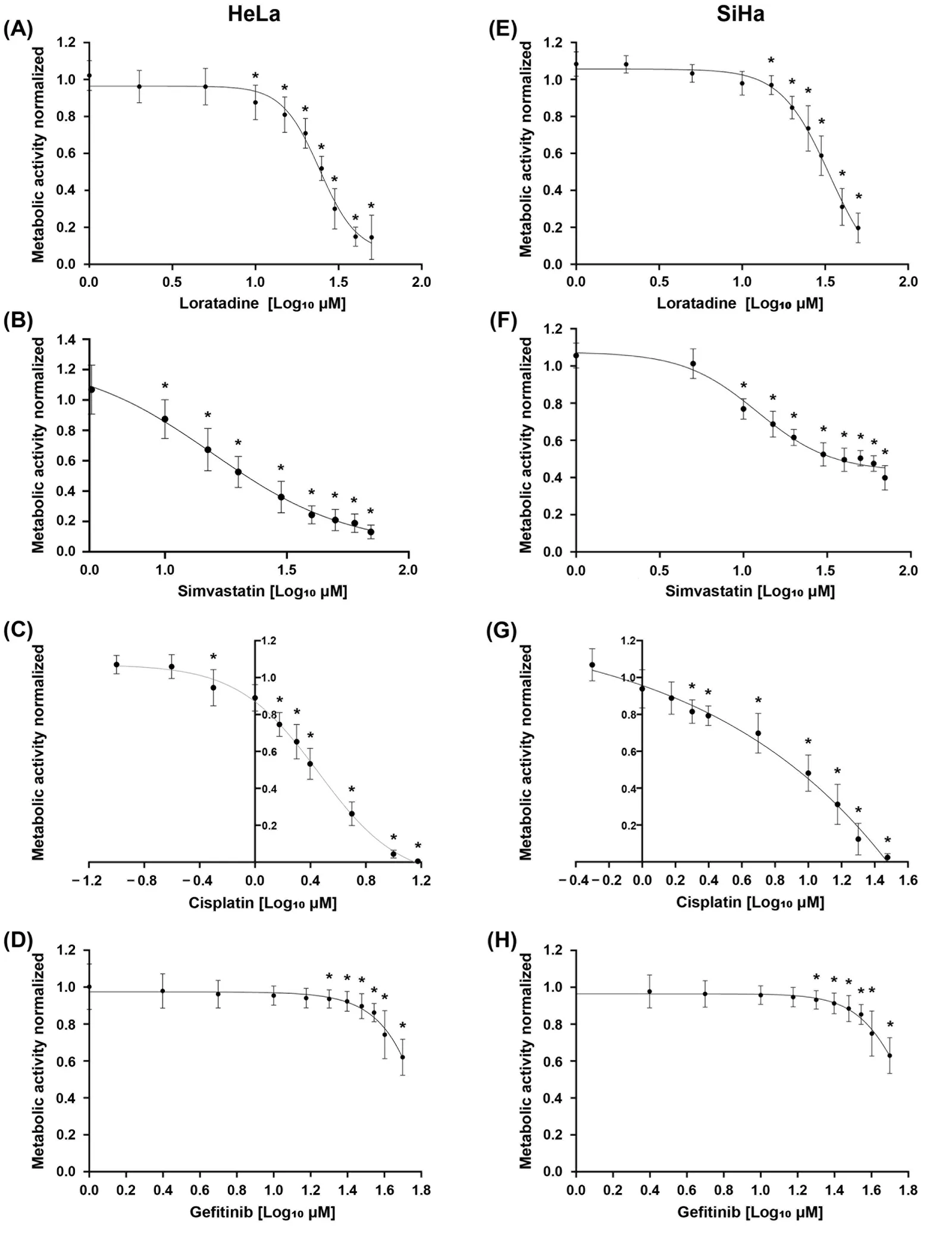
Effects of loratadine, simvastatin, cisplatin, and gefitinib monotherapies on the metabolic activity of HeLa (**A**–**D**) and SiHa (**E**–**H**) cells. Metabolic activity was evaluated using the MTT assay after 72 h of treatment with loratadine (1–50 µM), simvastatin (10–70 µM), cisplatin (HeLa: 0.10–15 µM; SiHa: 0.50–30 µM), and gefitinib (2.5–50 µM). Data were normalized to the vehicle-treated group and are presented as mean ± SD of three independent experiments with at least eight technical replicates each (black dots). * p < 0.05 vs. vehicle-treated control.

Cellular viability was evaluated by measuring the reduction in metabolic activity induced by each drug as monotherapy using the MTT assay in HeLa and SiHa CC cells. All drugs induced a concentration-dependent decrease in metabolic activity in both cell lines (Figure 1). Simvastatin and cisplatin exhibited the strongest inhibitory effects, whereas gefitinib showed a moderate reduction in metabolic activity at higher concentrations. IC_20_ and IC_50_ values were obtained from these concentration–response curves and are shown in Table 1. It is worth mentioning that the inhibitory effect of loratadine supports its potential as a repurposing antineoplastic agent. We then performed the drug combination experiments using the calculated IC values.

### 3.2. Drug Combinations Potentiate Reductions in Metabolic Activity in Cervical Cancer Cell Lines

**Figure 2:**
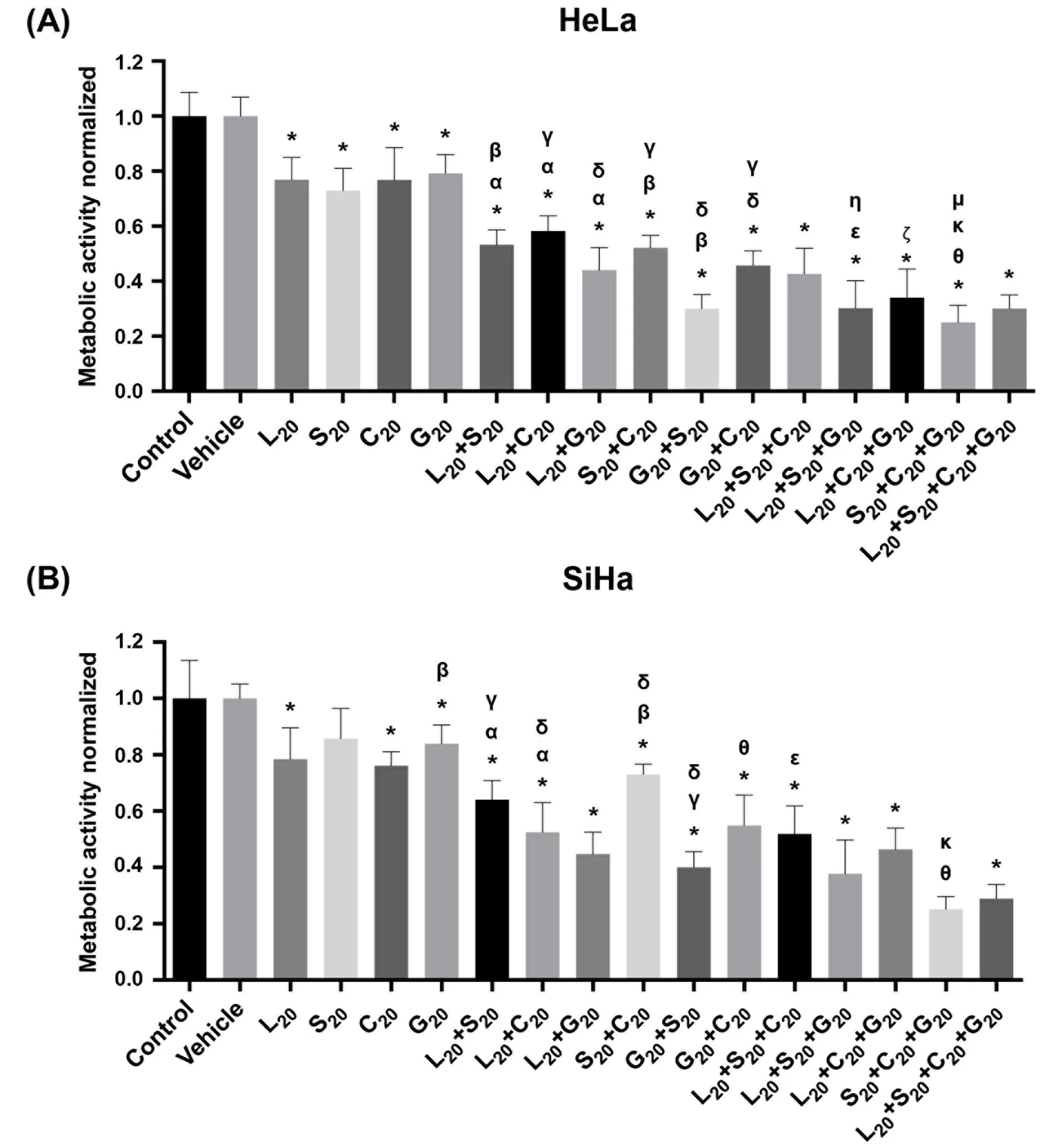
Effect of combination treatments on the metabolic activity of HeLa (**A**) and SiHa (**B**) cells. Cells were treated for 72 h with loratadine (L_20_), simvastatin (S_20_), cisplatin (C_20_), and gefitinib (G_20_) at their respective IC_20_, or the corresponding double, triple, and quadruple combinations at IC_2o’s_. Metabolic activity was evaluated by MTT assay. Data were normalized to the vehicle-treated group (Vh.) and are presented as mean ± SD from three independent experiments with at least eight technical replicates each. Statistical significance was determined at * *p* < 0.05 vs. vehicle-treated control; vs. L_20_ (α); vs. S_20_ (β); vs. C_20_ (γ); and vs. G_20_ (δ). In triple and quadruple combination treatments, significant differences were also observed vs. L_20_+S_20_ (ε); vs. L_20_+G_20_ (η); vs. L_20_+C_20_ (ζ); vs. S_20_+C_20_ (θ); vs. C_20_+G_20_ (κ); and vs. L_20_+S_20_+G_20_ (μ).

Drug combinations at their individual IC_20_ significantly reduced metabolic activity compared with monotherapies in both cell lines (Figure 2). In both cell lines, the most potent double combinations were loratadine–gefitinib and gefitinib–simvastatin, while the most effective triple combinations were loratadine–gefitinib–simvastatin and simvastatin–cisplatin–gefitinib. Notably, double and one of these triple combinations had an effect similar to the quadruple combination, suggesting that novel treatment options potentially avoid cisplatin side effects.

**Figure 3:**
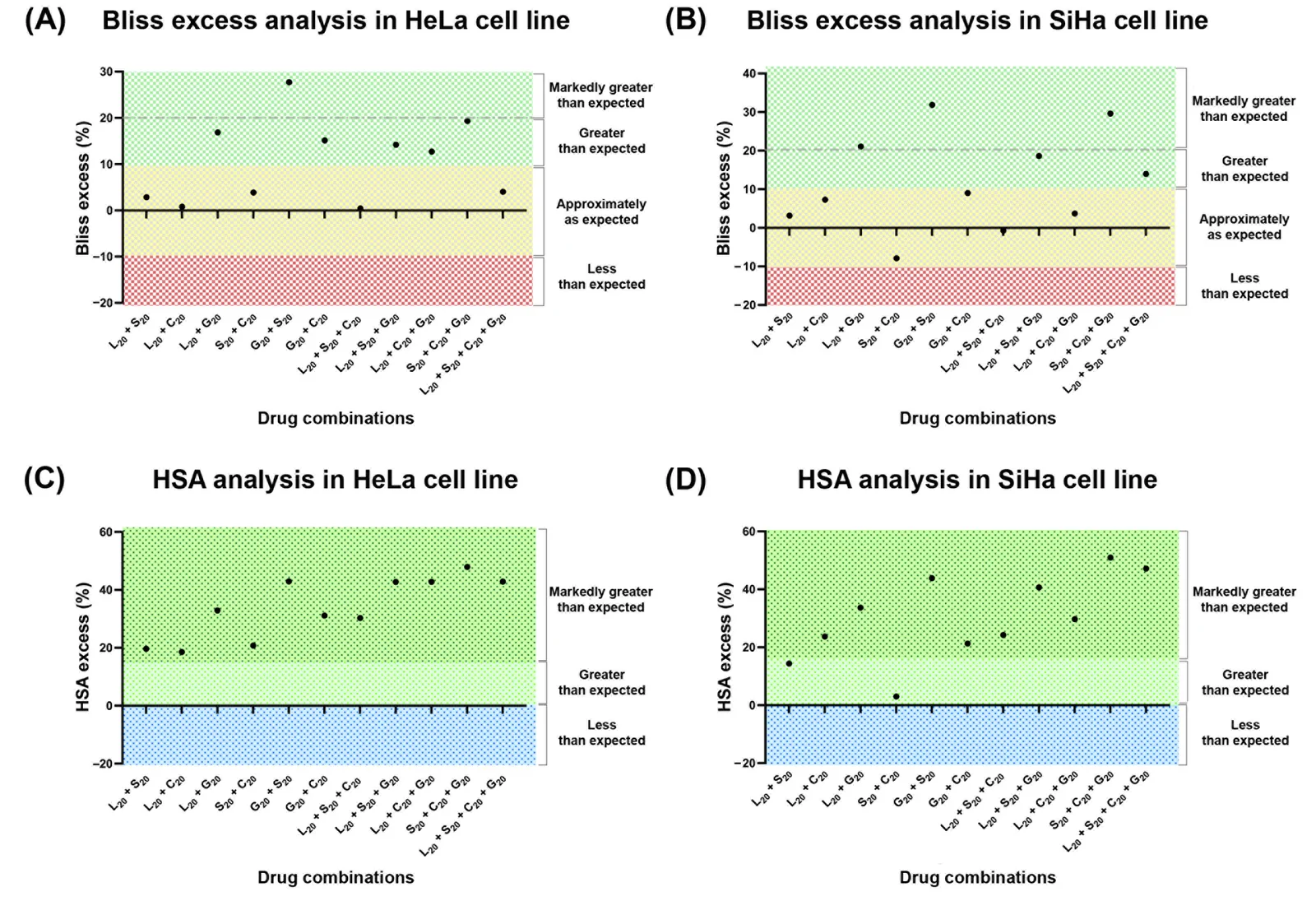
Drug interaction analysis using the Bliss Independence and Highest Single Agent (HSA) models in cervical cancer cell lines. (**A**) Bliss analysis in HeLa cells. (**B**) Bliss analysis in SiHa cells. (**C**) HSA analysis in HeLa cells. (**D**) HSA analysis in SiHa cells. Drug interactions were evaluated after 72 h of treatment based on metabolic activity inhibition. Bliss excess and HSA scores represent the difference between observed and expected effects, with positive, near-zero, and negative values indicating greater-than-expected, additive, and lower-than-expected interactions, respectively. Black dots indicate the percentage values of Bliss and HSA excess for each combination shown on the X-axis. Complete numerical results are provided in the Supplementary Material.

Bliss and HSA analyses [31,32] further supported these findings. The Bliss model showed that most combinations produced greater-than-expected inhibitory effects, and the HSA analysis showed positive HAS excess values for all combinations, indicating inhibitory effects exceeding those of the most active single agent. The highest interaction scores observed were for the combinations gefitinib–simvastatin, loratadine–simvastatin–gefitinib, simvastatin–cisplatin–gefitinib, and the quadruple regimen (Figure 3). Detailed numerical values are provided in Tables S1 and S2 in the Supplementary Materials.

### 3.3. Combination Treatments Increase Apoptosis in CC Cells

**Figure 4:**
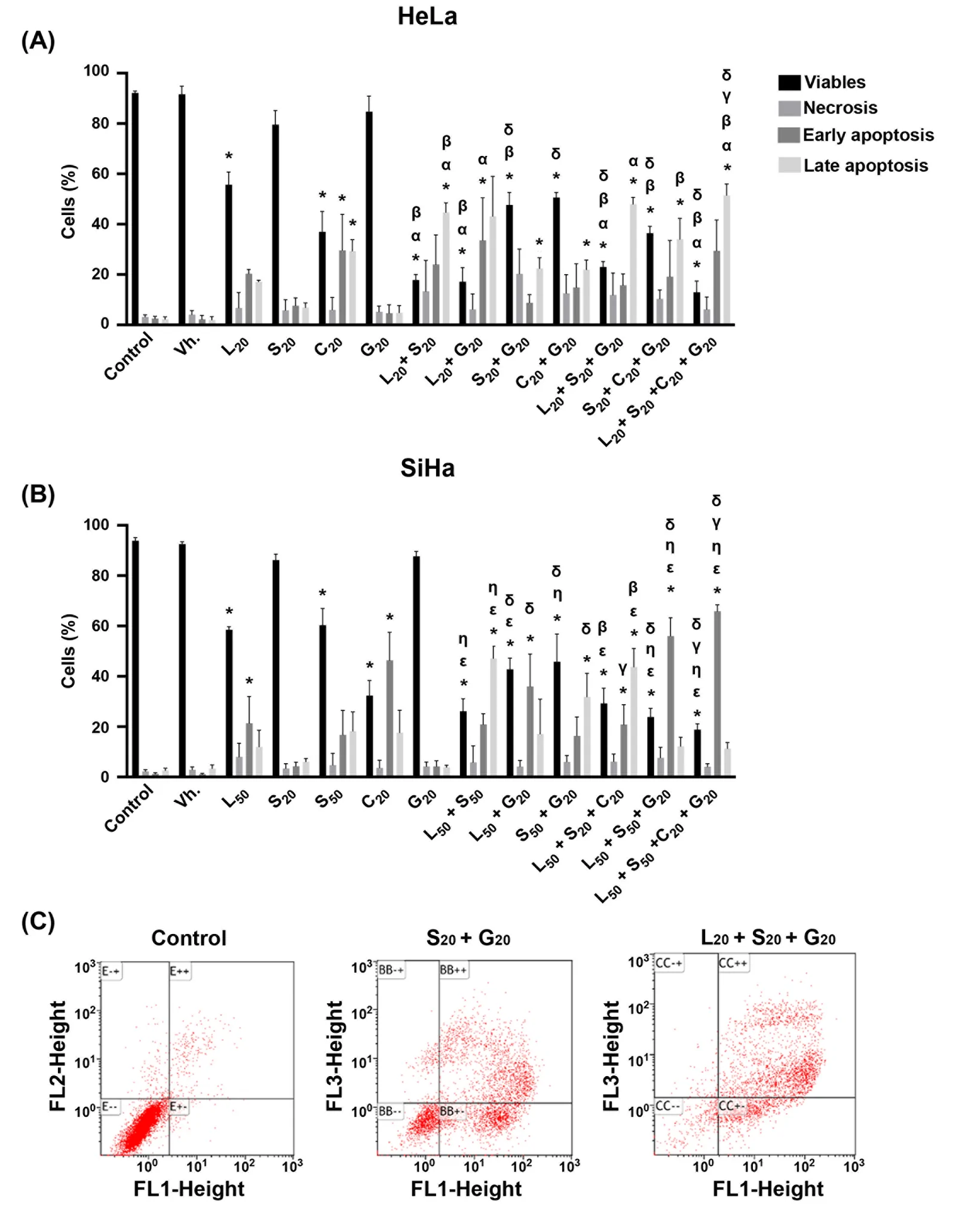
Induction of apoptosis in HeLa (**A**) and SiHa (**B**) cells by loratadine, simvastatin, cisplatin, and gefitinib as monotherapies and combination treatments. Cells were cultured for 72 h in the presence of loratadine (L), simvastatin (S), cisplatin (C), or gefitinib (G) at its respective inhibitory concentration (IC_20_ or IC_50_), either alone or in combination, or vehicle (Vh). Apoptosis was evaluated by Annexin V-FITC/PI flow cytometry. Results are presented as the mean ± SD of three independent experiments with three technical replicates each. Representative flow cytometry dot plots from control and treated SiHa cells are shown in panel (**C**). (*), (α), (β), (γ), (δ), (ε), and (η) indicate significant differences vs. vehicle-treated control, L_20_, S_20_, C_20_, G_20_, L_50_, and S_50_, respectively, *p* < 0.05.

Drug combination treatments significantly increased early and late apoptosis compared with monotherapies in both cell lines (Figure 4). Triple loratadine–simvastatin–gefitinib and the quadruple combination were similarly effective, each inducing the highest apoptotic responses and decreases in the percentage of viable cells. Representative Annexin V-FITC/PI dot plots for all treatments, monotherapies and combinations, are presented in Supplementary Figures S1 (SiHa) and S2 (HeLa).

### 3.4. Drug Combinations Reduce Clonogenic Survival and Cell Migration

**Figure 5:**
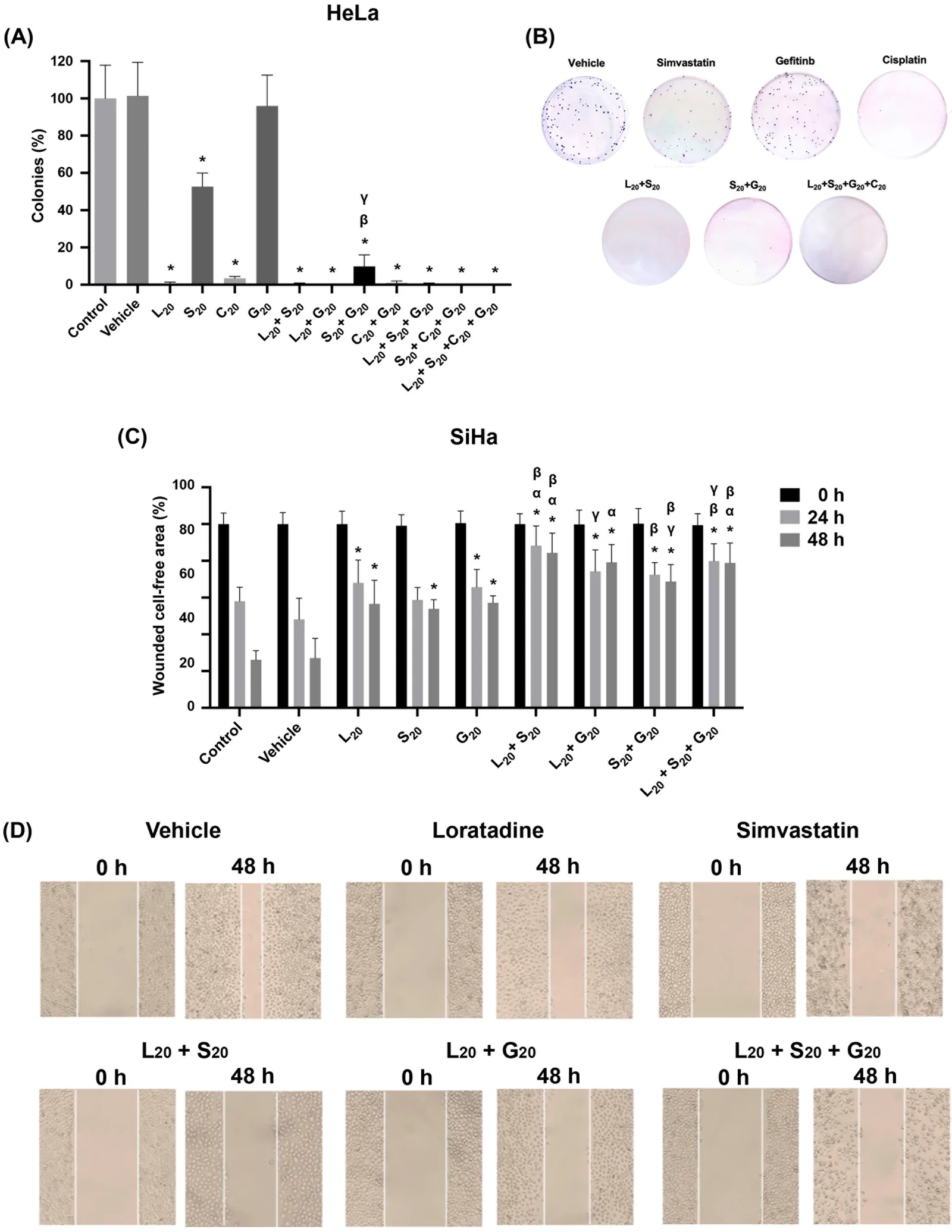
Effects of monotherapies and drug combination treatment on clonogenic survival and migratory ability of CC cells. (**A**) Effect of loratadine (L_20_), simvastatin (S_20_), cisplatin (C_20_), gefitinib (G_20_), and their combinations on colony formation in HeLa cells. Cells were treated for 72 h with IC_20_ concentrations, and clonogenic survival was evaluated by a colony formation assay after seven days in drug-free medium. Representative images of colony formation assays are shown in panel (**B**). (**C**) Effects of monotherapies and combination treatments on SiHa cell migration in a wound healing assay. Cells were treated with IC_20_ concentrations of loratadine, simvastatin, and gefitinib, individually or in combination, at 0, 24, and 48 h. Representative images of wound closure are shown in panel (**D**). Results are presented as mean ± SD from four independent experiments with four technical replicates each. * *p* < 0.05 vs. vehicle-treated control; vs. L_20_ (α); vs. S_20_ (β); and vs. G_20_ (γ).

Loratadine and cisplatin abolished the clonogenic capacity of HeLa cells. Interestingly, simvastatin produced only partial inhibition, and gefitinib had no effect, but when these drugs were combined, colony formation was almost completely inhibited. The remaining drug combinations abolished clonogenic ability completely (Figure 5A,B). Cisplatin toxicity and side effects in cancer patients are very well known. Here, we observed that drug combinations in the absence of cisplatin showed significant effects on cell metabolic activity, apoptosis, and clonogenic survival, which may be of clinical interest. Therefore, the remaining experiments were performed in the absence of cisplatin. Wound-healing assays showed that some double and triple loratadine–simvastatin–gefitinib combinations in the absence of cisplatin significantly reduced the cell migration of SiHa cells compared with controls and monotherapies (Figure 5C,D). 

Under our experimental conditions (see Section 2), it was not possible to grow colonies from single SiHa cells, probably because the number of cultured cells necessary to ensure growth from individual separated cells was too low. Similarly, cell migration experiments with HeLa cells were not performed because of poor cell attachment under the specific experimental conditions. Finally, we investigated the effect of drug combinations on tumor formation and cancer cell spreading in vivo.

### 3.5. In Vivo Tumor Formation and Dissemination Are Decreased by Combination Treatments

**Figure 6:**
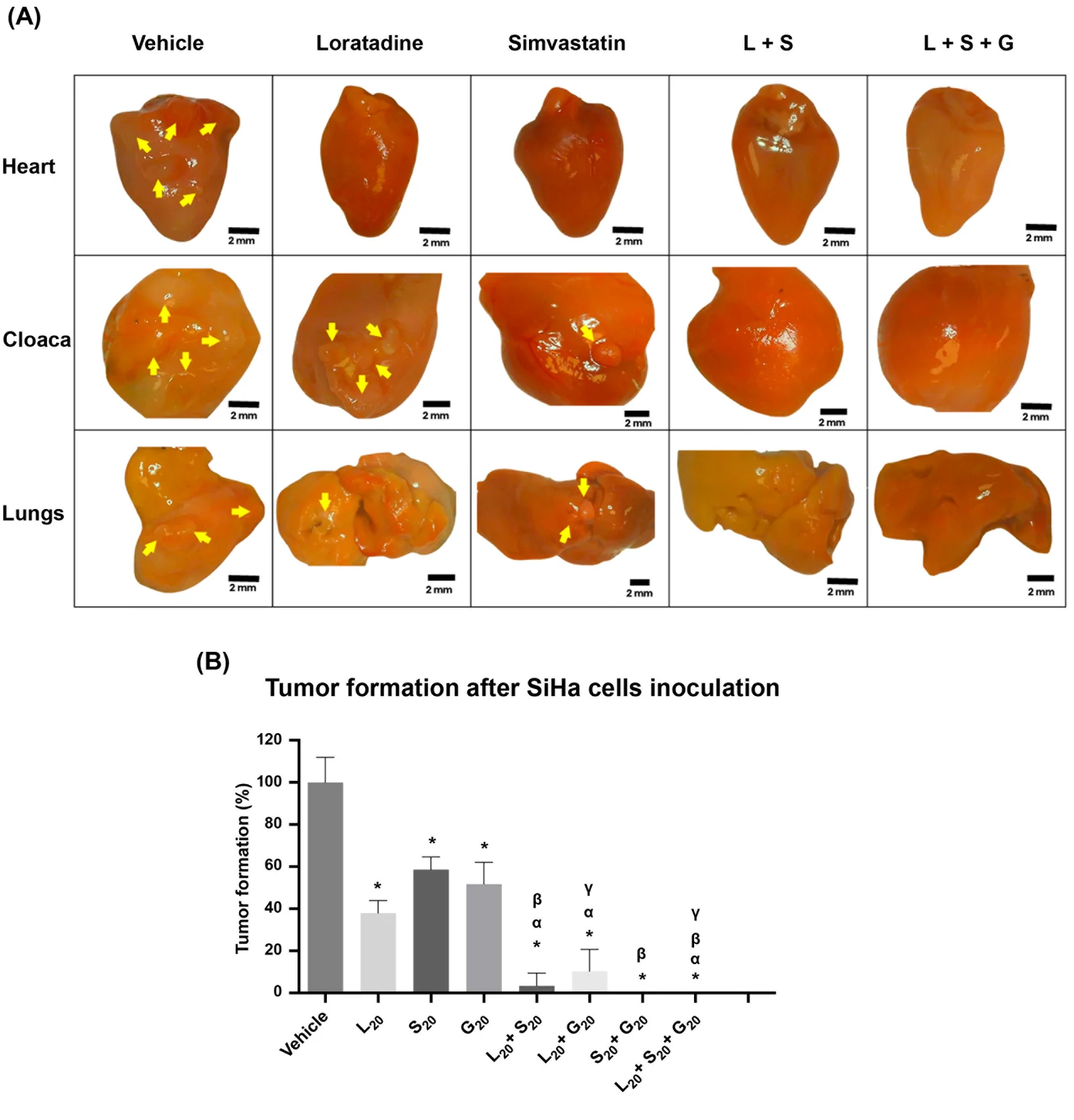
Effects of single-drug and combination treatments on tumor formation and spreading in vivo. (**A**) Representative images of tumor formation in the heart, cloaca, and lungs from embryos injected with SiHa cells treated with vehicle, loratadine (L), Simvastatin (S), and gefitinib (G) administered either as monotherapies or in double or triple combinations. Yellow arrows indicate visible tumor nodules. (**B**) Percentage of tumor formation calculated from the total number of tumors observed. Results are presented as mean ± SD from three independent experiments with two or three technical replicates each. Statistical significance (*p* < 0.05) was determined vs. vehicle-treated control (*); vs. L_20_ (α); vs. S_20_ (β); and vs. G_20_ (γ).

The antitumoral potential of the investigated drugs was evaluated using the CAM model. The vehicle-treated group developed tumor nodules in several organs, such as the heart, cloaca, and lungs (Figure 6A). Monotherapies clearly decreased tumor formation, while the double and triple combinations either almost abolished or completely inhibited tumor formation and metastatic dissemination (Figure 6B). Table and representative images of all experimental groups are included in the Supplementary Figure S3.

## 4. Discussion

In the present study, some two- or three-drug combinations inhibited metabolic activity, induced apoptosis, reduced clonogenic survival and migratory capacity, and decreased in vivo tumor nodule formation and spreading in CC models. These antineoplastic effects were observed even in the absence of cisplatin, suggesting novel treatment options that could potentially avoid cisplatin side effects. In fact, the enhanced inhibitory effects of drug combinations on metabolic activity were further supported by Bliss independence and HSA analyses. These findings demonstrated that several combinations, including those in the absence of cisplatin, produced greater-than-expected inhibition of cell metabolic activity in both cell lines, providing further support for the therapeutic potential of such combinations.

**Figure 7:**
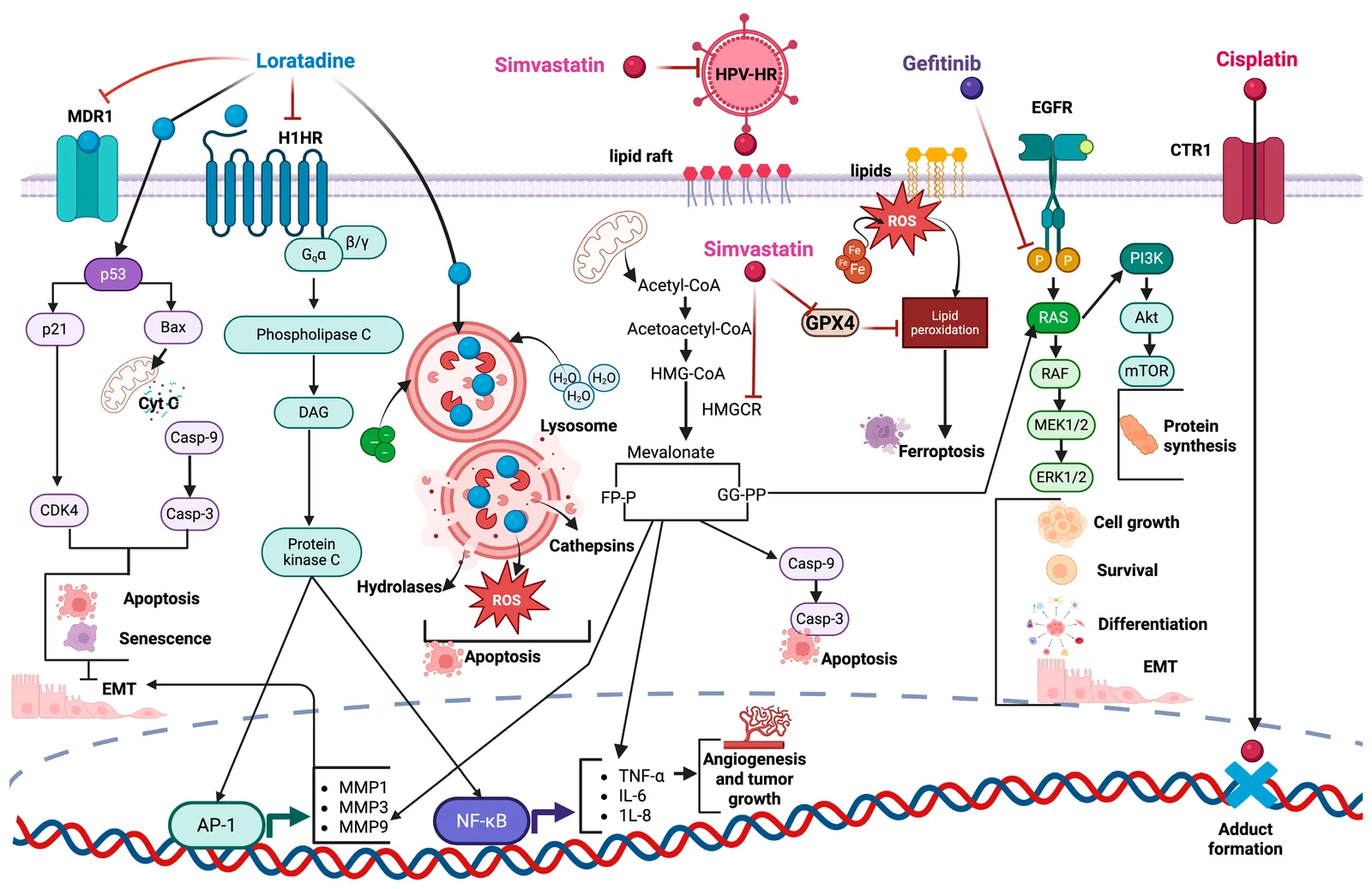
Potential mechanisms underlying the antitumor effects of loratadine, simvastatin, cisplatin, and gefitinib combinations. Loratadine blocks histamine H1 receptors (H1HR), activating proapoptotic pathways via phospholipase C and protein kinase C. This subsequently inhibits transcription factors such as AP-1, which is associated with metastatic processes involved in inflammatory signaling. Additionally, loratadine inhibits the MDR1 transporter. Simvastatin inhibits the enzyme HMG-CoA reductase, reducing the production of cholesterol intermediates such as farnesyl pyrophosphate (FP-P) and geranylgeranyl pyrophosphate (GG-PP), which are required for the isoprenylation of small GTPases such as Ras, thereby decreasing cell growth and survival. Gefitinib inhibits the epidermal growth factor receptor (EGFR) signaling, blocking activation of the RAS/RAF/MEK/ERK and PI3K/AKT/mTOR pathways, resulting in reduced cell proliferation and increased apoptosis, as well as suppression of epithelial–mesenchymal transition (EMT). Cell migration and invasion may be inhibited by the drug’s effects on EMT and metalloproteinase synthesis. Cisplatin, after internalization through the CTR1 transporter, induces DNA damage and activates apoptotic pathways. Interestingly, the convergence of single-drug effects may be potentiated in combined treatments. Created in: https://BioRender.com.

Reported mechanistic studies of the drugs investigated here allow us to suggest that diverse molecular mechanisms likely contribute to the antineoplastic effects of these drug combinations. Although single-drug mechanisms may act independently, several intracellular pathways may converge to enhance the anticancer activity of drug combinations (Figure 7).

Loratadine modulates key oncogenic signaling pathways, including STAT3, JNK, and p38 [26]. In retrospective studies, loratadine use improved survival outcomes in patients with lung and breast carcinoma [26,34]. The potentiated effect observed with the combination of loratadine and gefitinib supports previous findings of synergistic interactions between antihistamines and receptor tyrosine kinase (RTK) inhibitors, such as ibrutinib, emphasizing the significance of combination therapeutic strategies [35].

Persistent HR-HPV infection is the main risk factor for CC; thus, the antiviral properties of statins such as simvastatin may be particularly relevant. Statins can modulate antiviral immune responses and alter signaling pathways involved in viral infection, including JAK/STAT signaling [36]. In addition, statins alter the cell membrane composition by reducing cholesterol levels in lipid rafts, thereby disrupting lipid raft-dependent signaling pathways that regulate cell proliferation, survival, and migration. Together with the inhibition of the mevalonate pathway and protein prenylation, these mechanisms may contribute to the antineoplastic activity observed in HPV-transformed CC cells [37,38].

Although gefitinib showed limited effects on apoptosis induction and colony formation, its combination with loratadine or simvastatin significantly increased these effects. EGFR transphosphorylation is inhibited by gefitinib, whereas simvastatin suppresses the isoprenylation of small GTPases such as Ras. These two drugs simultaneously target the oncogenic Ras/Raf/MEK/ERK, Akt, and mTOR pathways, which regulate cell proliferation, survival, differentiation, metastasis, and protein synthesis [39,40,41]. This simultaneous inhibition may therefore contribute to the enhanced antitumor activity observed with this combination.

Loratadine has been shown to disrupt lysosomal membrane integrity, promoting cathepsin release and inducing reactive oxygen species (ROS) accumulation, leading to lysosome-dependent cell death [17,29,35,42,43], whereas simvastatin induces ferroptosis [16]. These differential roles may explain why cell death is potentiated by the combination of loratadine and simvastatin, highlighting the role of oxidative stress in their cytotoxicity and supporting their combined use in redox-targeted anticancer strategies.

Cisplatin is a first-line chemotherapeutic agent for multiple solid cancers, including CC, but acquired resistance and severe adverse effects limit its use. In the present study, we observed that the combination of cisplatin with simvastatin and gefitinib at their respective IC_20_ produced a significant inhibitory effect on metabolic activity in both cell lines. These combinations may help to overcome chemoresistance. In line with this proposal, gefitinib has been shown to enhance cisplatin sensitivity by inhibiting EGFR signaling in resistant NSCLC cell lines [44].

Although some of the drug concentrations investigated here exceeded clinically achievable plasma levels, they were selected to investigate concentration-dependent biological effects under continuous 72 h in vitro exposure, which does not directly replicate the intermittent pharmacokinetic profiles observed in patients. However, observational epidemiological and clinical studies with single drugs or combinations in different cancers [11,12,13,14,23,24,34,43] strengthen the in vitro observations. Therefore, the pharmacokinetic properties of these compounds in combination, including their tissue distribution, should be further investigated in future in vivo studies using clinically relevant dosing.

Loratadine and cisplatin abolished the clonogenic capacity of CC cells as monotherapies and in combination with other drugs. This finding is particularly relevant because clonogenic survival is associated with tumor recurrence and therapeutic resistance.

Many cancer patients die because of metastasis. Interestingly, in the absence of cisplatin, the triple-drug combination of gefitinib, simvastatin, and loratadine inhibited the cells’ capacity to migrate, form nodules, and disseminate, supporting its potential to impair the invasive behavior of CC cells. These drugs may interfere with pathways involved in tumor progression and metastatic dissemination by targeting EGFR, Ras/Rho signaling, EMT-related and apoptosis mechanisms, and MMP secretion [17,26,30]. As depicted in Figure 7, the convergence of single-drug effects may be potentiated in combined treatments.

Recent advances in nanotechnology, including nanoparticle-based delivery systems and nanovaccines, may further enhance the efficacy and tumor targeting of the proposed drug combinations while reducing off-target toxicity, representing a promising avenue for future translational studies [45,46].

The drugs evaluated here are widely used in clinical practice and have well-established pharmacological and safety profiles, supporting their suitability for drug repurposing studies. Nonetheless, some limitations should be addressed in future work. These include the use of non-cancerous cervical epithelial cells and cervical cancer in vivo models that more closely resemble disease development in the cervical tissue of adult organisms, which would also enable pharmacokinetic and toxicity evaluation of the drug combinations. Moreover, mechanistic validation studies, such as pathway rescue experiments and siRNA knockdown, are needed to precisely elucidate the molecular mechanisms involved in the synergistic effects of the drug combinations. Noteworthy, future investigations using patient-derived models, as well as long-term resistance studies, would also strengthen the translational potential of our findings.

## 5. Conclusions

The combination of loratadine, simvastatin, and gefitinib, even in the absence of cisplatin, displayed significant anticancer activity in vitro and in vivo in CC cell lines. The relative safety of these drugs makes them strong candidates for repurposing and combination as a promising therapeutic approach for the benefit of CC patients. This strategy may help improve efficacy and overcome the resistance observed with common chemotherapeutic agents such as cisplatin.

## Figures and Tables

**Table 1:** Inhibitory concentrations (ICs) of loratadine, simvastatin, cisplatin, and gefitinib obtained from metabolic activity assays in cervical cancer cell lines.

Drug	HeLa		SiHa	
	IC_20_ [µM]	IC_50_ [µM]	IC_20_ [µM]	IC_50_ [µM]
Loratadine	16.9	24.7	20.0	28.2
Simvastatin	7.8	16.5	7.9	14.1
Cisplatin	1.1	2.9	2.5	10.0
Gefitinib	14.3	19.9	20.0	35.6

## Data Availability

The datasets generated and/or analyzed during the current study are available in the Supplementary Materials or from the corresponding author upon reasonable request.

## Supplementary Materials

The following supporting information can be downloaded at: https://www.mdpi.com/article/10.3390/cancers18162589/s1, Figure S1. Representative Annexin V-FITC/PI dot plots showing apoptosis analysis in SiHa cells following treatment with vehicle, monotherapies, and combination regimens. Quadrants indicate viable (Annexin V−/PI−), early apoptotic (Annexin V+/PI−), late apoptotic (Annexin V+/PI+), and necrotic (Annexin V−/PI+) cell populations. Abbreviations: Vh, vehicle; L, loratadine; S, simvastatin; G, gefitinib; C, cisplatin. Figure S2. Representative Annexin V-FITC/PI dot plots showing apoptosis analysis in HeLa cells following treatment with vehicle, monotherapies, and combination regimens. Quadrants indicate viable (Annexin V−/PI−), early apoptotic (Annexin V+/PI−), late apoptotic (Annexin V+/PI+), and necrotic (Annexin V−/PI+) cell populations. Abbreviations: Vh, vehicle; L, loratadine; S, simvastatin; G, gefitinib; C, cisplatin. Figure S3. Treatment groups (upper Table) and representative images of metastatic tumor nodules in the heart, cloaca, and lungs from the chick chorioallantoic membrane (CAM) assay. Representative organs are shown for the groups: sham, vehicle, monotherapy, and combination treatment groups. Abbreviations: Vh, vehicle; L, loratadine; S, simvastatin and G, gefitinib. Table S1. Determinations of pharamcological interactions in HeLa cell line. Table S2. Determinations of pharmacological interactions in SiHa cell line.

## Abbreviations

Akt	Protein kinase B
ANOVA	Analysis of variance
Ara-C	Cytarabine
ATCC	American Type Culture Collection
BTK	Bruton’s tyrosine kinase
CAM	Chick chorioallantoic membrane
CC	Cervical cancer
CO_2_	Carbon dioxide
DMSO	Dimethyl sulfoxide
DMEM	Dulbecco’s Modified Eagle Medium
DNA	Deoxyribonucleic acid
EGFR	Epidermal growth factor receptor
EMT	Epithelial–mesenchymal transition
ERK	Extracellular signal-regulated kinase
FBS	Fetal bovine serum
FITC	Fluorescein isothiocyanate
HPV	Human papillomavirus
HPV-HR	High-risk human papillomavirus
IC_20_	20% inhibitory concentration
IC_50_	50% inhibitory concentration
JAK/STAT	Janus kinase/signal transducer and activator of transcription
JNK	c-Jun N-terminal kinase
MMPs	Matrix metalloproteinases
mTOR	Mammalian target of rapamycin
MTT	3-(4,5-dimethylthiazol-2-yl)-2,5-diphenyltetrazolium bromide
NSCLC	Non-small cell lung cancer
PBS	Phosphate-buffered saline
PI	Propidium iodide
Raf	Rapidly accelerated fibrosarcoma kinase
Ras	Rat sarcoma
ROS	Reactive oxygen species
RTK	Receptor tyrosine kinase
SD	Standard deviation
SPF	Specific pathogen-free
STAT3	Signal transducer and activator of transcription 3
