# Supplementary material for: Antineoplastic Activity of the Combination Loratadine–Simvastatin–Gefitinib in HPV-Positive Cervical Cancer Cells: In Vitro and In Vivo Studies
Source: Cancers (Basel). 2026 Aug 12;18(16):2589. doi: 10.3390/cancers18162589 (PMC13511572; doi:10.3390/cancers18162589)
Supplement: Supplementary file 1 [file cancers-18-02589-s001.zip › cancers-4433629-supplementary.pdf]

Supplementary Materials

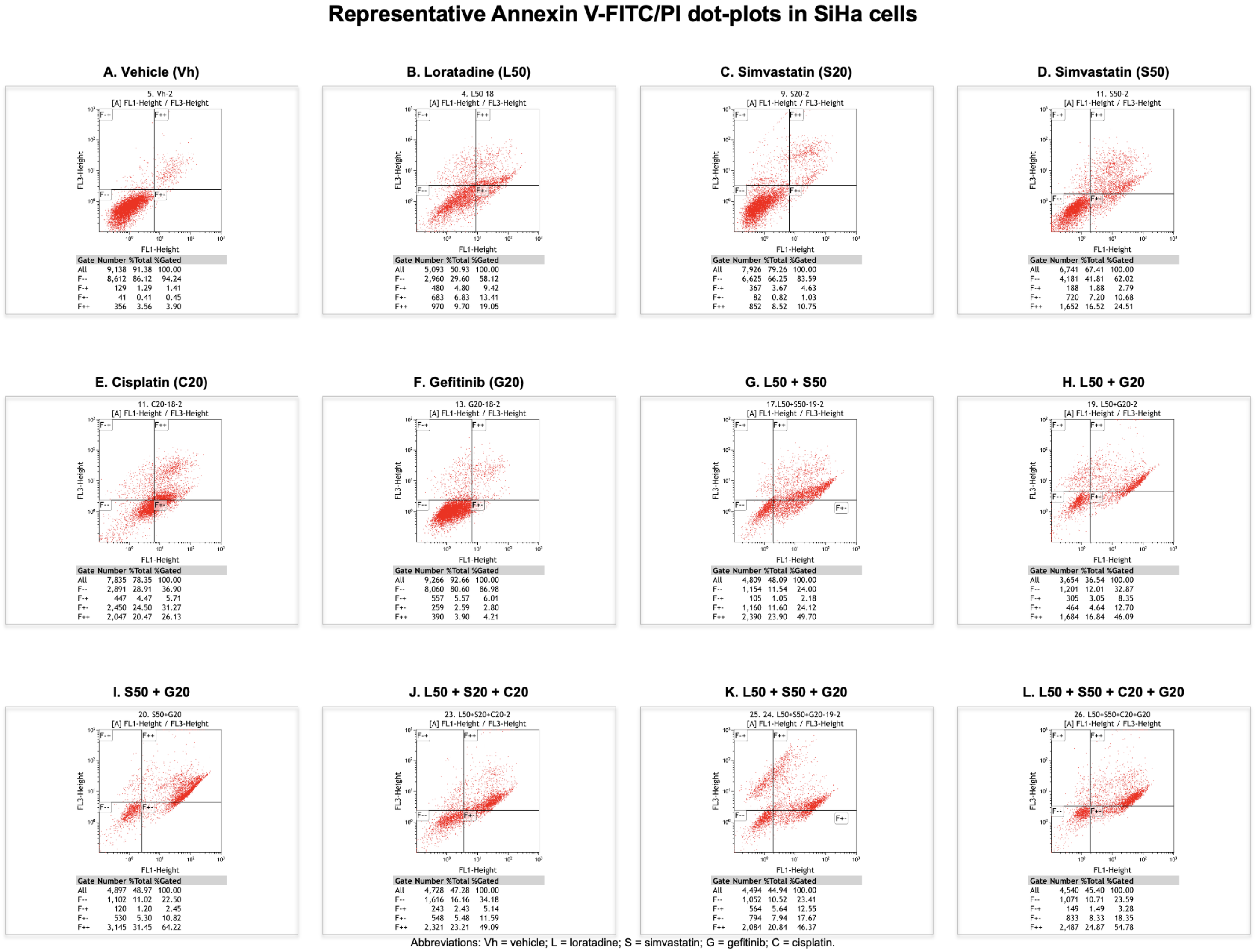

Figure S1. Representative Annexin V-FITC/PI dot plots showing apoptosis analysis in SiHa cells following treatment with vehicle, monotherapies, and combination regimens. Quadrants indicate viable (Annexin V−/PI−), early apoptotic (Annexin V+/PI−), late apoptotic (Annexin V+/PI+), and necrotic (Annexin V−/PI+) cell populations. Abbreviations: Vh, vehicle; L, loratadine; S, simvastatin; G, gefitinib; C, cisplatin.

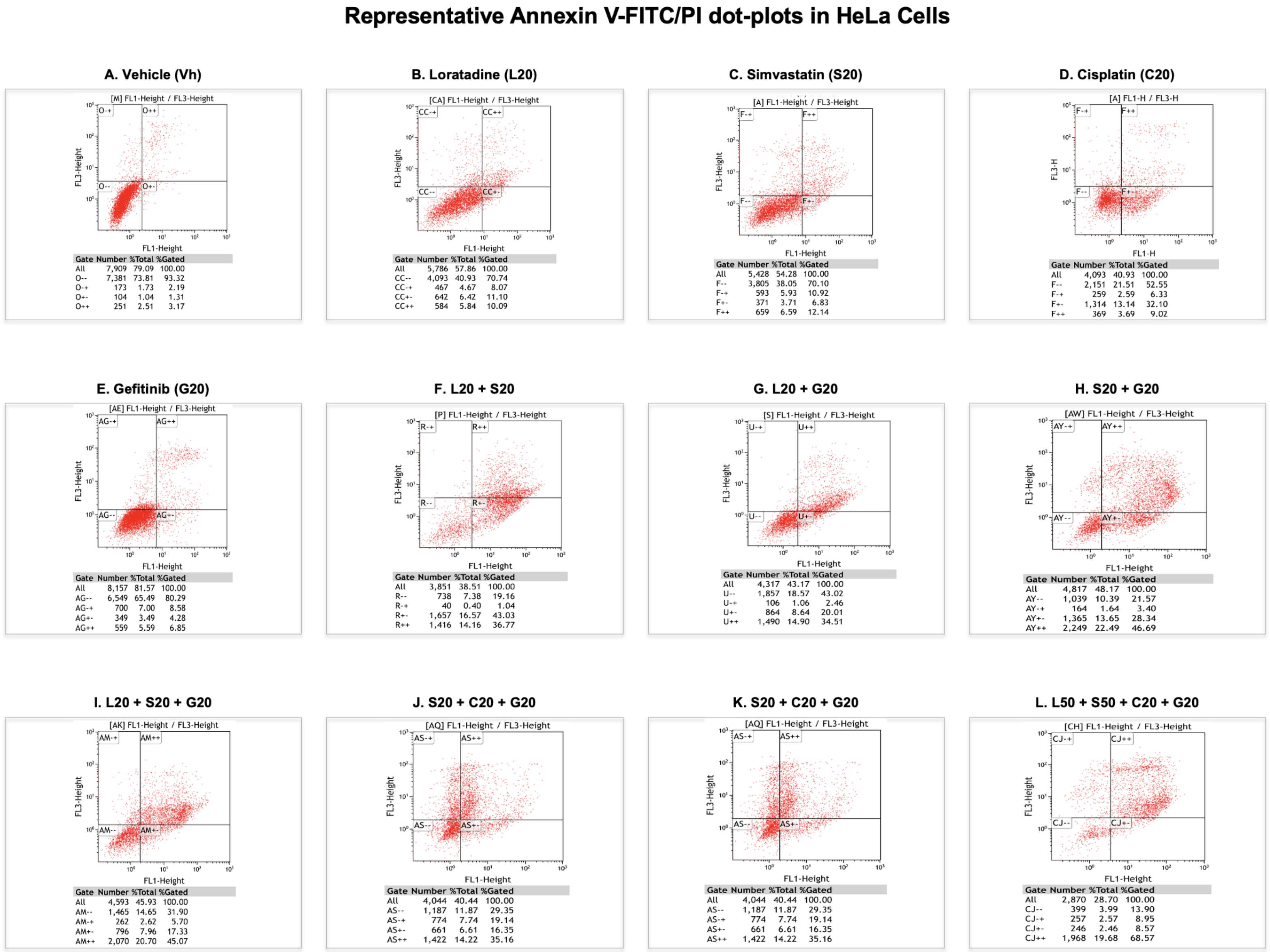

Abbreviations: Vh = vehicle; L = loratadine; S = simvastatin; G = gefitinib; C = cisplatin.

Figure S2. Representative Annexin V-FITC/PI dot plots showing apoptosis analysis in HeLa cells following treatment with vehicle, monotherapies, and combination regimens. Quadrants indicate viable (Annexin V−/PI−), early apoptotic (Annexin V+/PI−), late apoptotic (Annexin V+/PI+), and necrotic (Annexin V−/PI+) cell populations. Abbreviations: Vh, vehicle; L, loratadine; S, simvastatin; G, gefitinib; C, cisplatin.

Group	Treatment
1	Sham control (without cell inoculation or treatment)
2	Vehicle control (cell-inoculated, DMSO-treated)
3	Loratadine (L)
4	Simvastatin (S)
5	Gefitinib (G)
6	Loratadine + Simvastatin (L + S)
7	Loratadine + Gefitinib (L + G)
8	Simvastatin + Gefitinib (S + G)
9	Loratadine + Simvastatin + Gefitinib (L + S + G)

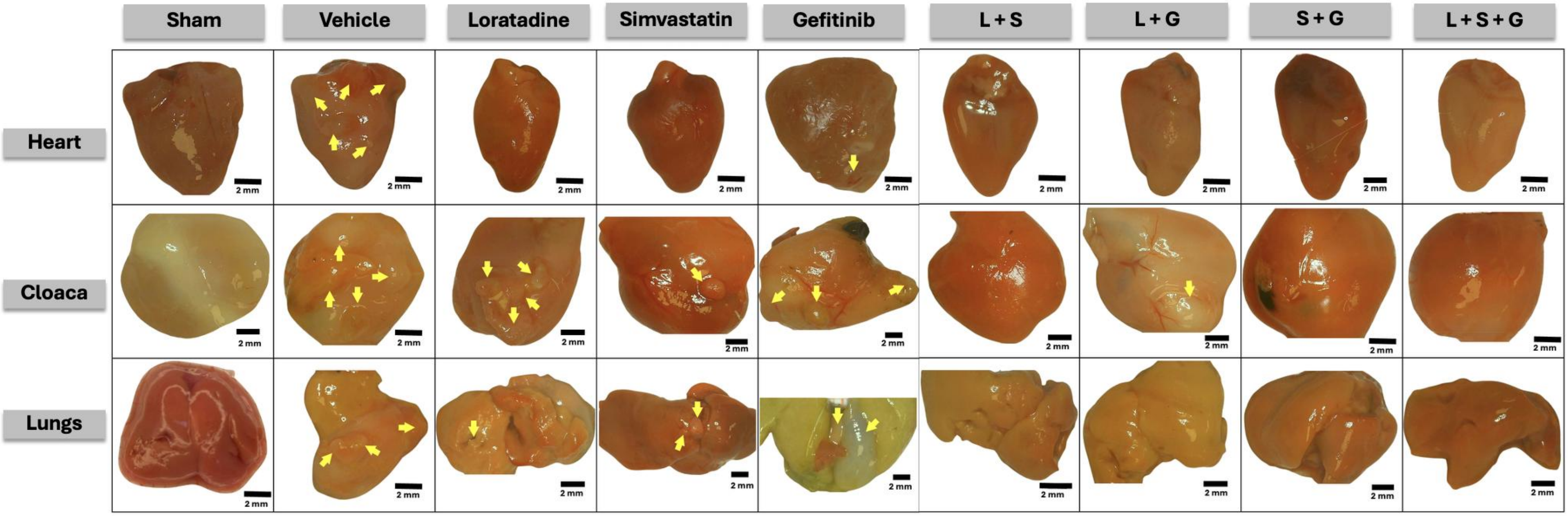

Figure S3. Treatment groups (upper Table) and representative images of metastatic tumor nodules in the heart, cloaca, and lungs from the chick chorioallantoic membrane (CAM) assay. Representative organs are shown for the groups: sham, vehicle, monotherapy, and combination treatment groups. Abbreviations: Vh, vehicle; L, loratadine; S, simvastatin and G, gefitinib.

Table S1. Determinations of pharamcological interactions in HeLa cell line

Bliss Independece

**Combinations**	**Expected Bliss Inhibition (%)**	**Observed Inhibition (%)**	**Bliss Excess (pp)**	**Interpretation**
$L_{20}+S_{20}$	43.92%	46.78%	2.85%	Greater than expected
$L_{20}+C_{20}$	40.93%	41.74%	0.81%	Greater than expected
$L_{20}+G_{20}$	**39.12%**	**55.97%**	**16.85%**	**Markedly greater than expected**
$S_{20}+C_{20}$	44.01%	47.89%	3.88%	Greater than expected
$G_{20}+S_{20}$	**42.30%**	**70.02%**	**27.72%**	**Markedly greater than expected**
$G_{20}+C_{20}$	**39.22%**	**54.33%**	**15.12%**	**Markedly greater than expected**
$L_{20}+S_{20}+C_{20}$	56.94%	57.38%	0.44%	Greater than expected
$L_{20}+S_{20}+G_{20}$	**55.62%**	**69.82%**	**14.20%**	**Markedly greater than expected**
$L_{20}+C_{20}+G_{20}$	53.25%	65.97%	12.72%	Markedly greater than expected
$S_{20}+C_{20}+G_{20}$	**55.68%**	**75.01%**	**19.33%**	**Markedly greater than expected**
$L_{20}+S_{20}+C_{20}+G_{20}$	65.91%	69.95%	4.03%	Greater than expected

Highest Single Agent (HSA) models

Individual Inhibitions	**Expected HSA Inhibition (%)**	**Observed Inhibition (%)**	**HSA Excess (percentage points)**	**Interpretation**
$L_{20}+S_{20}$	27.09%	46.78%	19.68%	Exceeded the most active single agent
$L_{20}+C_{20}$	23.20%	41.74%	18.54%	Exceeded the most active single agent
$L_{20}+G_{20}$	23.08%	55.97%	32.88%	**Exceeded the most active single agent**
$S_{20}+C_{20}$	27.09%	47.89%	20.80%	Exceeded the most active single agent
$G_{20}+S_{20}$	27.09%	70.02%	42.92%	**Exceeded the most active single agent**
$G_{20}+C_{20}$	23.20%	54.33%	31.13%	Exceeded the most active single agent
$L_{20}+S_{20}+C_{20}$	27.09%	57.38%	30.28%	**Exceeded the most active single agent**
$L_{20}+S_{20}+G_{20}$	27.09%	69.82%	42.72%	**Exceeded the most active single agent**
$L_{20}+C_{20}+G_{20}$	23.20%	65.97%	42.77%	**Exceeded the most active single agent**
$S_{20}+C_{20}+G_{20}$	27.09%	75.01%	47.92%	**Exceeded the most active single agent**
$L_{20}+S_{20}+C_{20}+G_{20}$	27.09%	69.95%	42.85%	**Exceeded the most active single agent**

Table S2. Determinations of pharmacological interactions in SiHa cell line

Combinations	Expected Bliss Inhibition (%)	Observed Inhibition (%)	Bliss Excess (percentage points)	Interpretation
**$L_{20} + S_{20}$**	32.80	35.95	3.15	Greater than expected
**$L_{20} + C_{20}$**	40.30	47.58	7.28	Greater than expected
**$L_{20} + G_{20}$**	**34.18**	**55.24**	**21.07**	**Markedly greater than expected**
**$S_{20} + C_{20}$**	34.80	26.91	-7.88	Lower than expected
**$G_{20} + S_{20}$**	**28.10**	**59.94**	**31.84**	**Markedly greater than expected**
**$G_{20} + C_{20}$**	36.13	45.15	9.02	Greater than expected
**$L_{20} + S_{20} + C_{20}$**	48.85	48.12	-0.73	Comparable to expected
**$L_{20} + S_{20} + G_{20}$**	**43.61**	**62.24**	**18.63**	**Markedly greater than expected**
**$L_{20} + C_{20} + G_{20}$**	49.90	53.61	3.70	Greater than expected
**$S_{20} + C_{20} + G_{20}$**	**45.28**	**74.87**	**29.59**	**Markedly greater than expected**
**$L_{20} + S_{20} + C_{20} + G_{20}$**	**57.08**	**71.06**	**13.98**	**Markedly greater than expected**

Highest Single Agent (HSA) models

Individual Inhibitions	**Expected HSA Inhibition (%)**	**Observed Inhibition (%)**	**HSA Excess (percentage points)**	**Interpretation**
L_{20}; S_{20}	21.56	35.95	14.39	**Exceeded the most active single agent**
L_{20}; C_{20}	23.89	47.58	23.69	**Exceeded the most active single agent**
L_{20}; G_{20}	21.56	55.24	33.68	**Exceeded the most active single agent**
S_{20}; C_{20}	23.89	26.91	3.02	Exceeded the most active single agent
G_{20}; S_{20}	16.08	59.94	43.86	**Exceeded the most active single agent**
G_{20}; C_{20}	23.89	45.15	21.26	**Exceeded the most active single agent**
L_{20}; S_{20}; C_{20}	23.89	48.12	24.23	**Exceeded the most active single agent**
L_{20}; S_{20}; G_{20}	21.56	62.24	40.68	**Exceeded the most active single agent**
L_{20}; C_{20}; G_{20}	23.89	53.61	29.72	**Exceeded the most active single agent**
S_{20}; C_{20}; G_{20}	23.89	74.87	50.98	**Exceeded the most active single agent**
L_{20}; S_{20}; C_{20}; G_{20}	23.89	71.06	47.17	**Exceeded the most active single agent**
